# A study of the relationship between cough and wheezing complicated by common respiratory viral infections in infants and secondary thrombocythemia

**DOI:** 10.1371/journal.pone.0326369

**Published:** 2025-07-09

**Authors:** Ping He, Fangqi Hu, Fei Wang

**Affiliations:** Department of Pediatrics, Anqing Municipal Hospital, Anqing, Anhui, China; University of Alcalá, SPAIN

## Abstract

**Objective:**

To explore the relationship between respiratory viral infections complicated by cough and wheezing and the clinical features of thrombocythemia in infants.

**Methods:**

We retrospectively analyzed the clinical data of 200 infants with fever, cough and wheezing who were admitted to the Department of Pediatrics of Anqing Municipal Hospital between January 2023 and October 2024. Respiratory viruses were detected using real-time PCR to screen for virus-positive infants. Infants were classified into the thrombocythemia group (ST group) and the non-thrombocythemia group (non-ST group) based on platelet counts. We compared the clinical characteristics of the two groups and analyzed the relationships between thrombocythemia, positive respiratory virus results, and cough with wheezing.

**Results:**

The overall virus positivity detection rate was 56.5% (113/200). In the ST group, patients with positive respiratory virus results had significantly longer hospital stays, higher fever incidence, and more severe cough symptoms (P < 0.05), and significantly higher leukocyte and interleukin-6 (IL-6) levels (P < 0.05). Among the virus-positive cases, the duration of fever, cough, and wheezing was longer in the ST group than in the non-ST group (P < 0.05). Higher platelet counts were significantly associated with longer hospitalization duration, prolonged cough duration and a higher co-occurring wheezing rate (p < 0.05). Point-biserial correlation analysis indicated that thrombocythemia was closely associated with respiratory virus positivity complicated by cough and wheeze (r = 0.314, P < 0.05). Multifactorial analysis revealed that age, non-respiratory symptoms, platelet levels, and IL-6 level were independent influencing factors for respiratory virus-positive infants with complicated cough and wheezing (P < 0.05). Receiver operating characteristic (ROC) curve analysis showed that the areas under the curve of thrombocythemia for assessing hospital stay duration and cough and wheeze durations were 0.964 and 0.936, respectively (P < 0.05), indicating a significant assessment value.

**Conclusion:**

Infant respiratory viral infections complicated by cough and wheezing are closely related to secondary thrombocythemia. Elevated platelet levels can prolong the hospital stay and cough duration. The clinical risk in children can be assessed early by measuring platelet levels.

## 1. Introduction

Respiratory viral infections are the most common type of disease in childhood, and children with viral infections can present with different clinical manifestations from mild common cold including fever, sore throat, acute otitis media, cough and extension to severe wheezing, especially in infancy, and account for approximately 75% of all pediatric inpatient admissions [1].

Thrombocythemia is a common condition in children and is generally referred to as thrombocythemia with a platelet count of >400 x 109/L [2]. Thrombocythemia can be categorized into primary thrombocythemia and secondary thrombocythemia (ST), where primary thrombocythemia mainly arises from primary bone marrow disorders, such as essential thrombocythemia, true erythrocytosis, chronic granulocytic leukemia, or myelodysplastic syndromes [3]. In contrast primary thrombocythemia has an increased risk of thrombosis and bleeding compared to secondary thrombocythemia [4]. In contrast, ST with elevated platelet levels is mostly a process caused by extrinsic factors, which can be attributed to transient processes such as acute blood loss, acute infections, or persistent forms of reactive thrombocythemia including iron deficiency, splenelessness, cancer, and chronic inflammatory or infectious diseases [2,5] with acute respiratory viral infections accounting for approximately 80% of the causes of ST in infants and children [6].

Cough and asthma in infants induced by viral infections are common diseases in pediatric outpatient and inpatient units, and pediatricians often observe that some children with respiratory viral infections complicated by cough and asthma often have elevated platelets for unknown reasons. Several studies have suggested that thrombocythemia may be an early marker of RSV and HRV infections [7], and it has been found that children with thrombocythemia associated with these viral infections have more severe symptoms of asthma [8]. However, the reason for the relationship between the two has not been elucidated; therefore this study aimed to analyze the correlation between respiratory viral infections with cough and wheezing and secondary thrombocythemia in infants.

## 2. Objects and methods

### 2.1 Study subjects

A total of 200 infants with fever and cough, hospitalized in the Department of Pediatrics of Anqing Municipal Hospital between January 2023 and October 2024, were selected as the study subjects. Among them, 105 were male and 95 were female, with ages ranging from 30 days to 12 months old. After admission, infants with serious underlying diseases such as congenital airway and gastrointestinal tract developmental anomalies, congenital heart disease, primary immunodeficiency, and primary thrombocythemia were excluded. This study was approved by the Institutional Review Board (IRB) of Anqing Municipal Hospital (approval number: 2023−087). Informed written consent was obtained from the parents/guardians of all infant participants, as documented in the consent forms archived at the hospital ethics office.

### 2.2 Research method

After admission, deep nasopharyngeal secretions were collected from 200 children with a fever and cough (The clinical data were provided by he Department of Pediatrics of Anqing Municipal Hospital and accessed for research purposes on December 10, 2024. The dataset included patient records from January 2023 and October 2024). Real-time PCR was performed to detect respiratory viruses, including respiratory syncytial virus (RSV), influenza A virus (IFV-A), influenza B virus (IFV-B), rhinovirus (HRV), parainfluenza virus (HPIV), adenovirus (ADV), and Epstein-Barr virus (EBV). The children were screened for viral infection. Based on the platelet count (PLT), the children were categorized into the thrombocythemia group (ST group, PLT > 400 × 10⁹/L) and the non-thrombocythemia group (non-ST group, PLT ≤ 400 × 10⁹/L). Peripheral blood white blood cell (WBC) count, hemoglobin level, C-reactive protein (CRP) level, and interleukin-6 (IL-6) level were measured within 24 hours of admission for all children.

### 2.3 Statistical treatment

SPSS software(version 16.0) was used for the statistical analysis. Measurement data are expressed as mean ± standard deviation (x ± s), and the t-test was used for between-group comparisons. Count data are expressed as frequency (n) and rate (%), and the chi-square test was used for between-group comparisons. Point-biserial correlation and logistic regression analyses were performed, and the ROC curve was used to assess the predictive value of thrombocythemia. Statistical significance was set at P < 0.05.

## 3. Results

### 3.1 Distribution of virus-positive and thrombocythemia cases

There were 83 virus-positive cases of thrombocythemia(ST group), including 45 males and 38 females, aged 1–12 months. Among them, 69 cases (83.1%) had mild thrombocythemia(400–699 × 10⁹/L), 11 cases (13.3%) had moderate-to-severe thrombocythemia(≥700 × 10⁹/L), and 3 cases (3.6%) had platelet counts > 1000 × 10⁹/L. There were 30 virus-positive cases with normal platelets (non-ST group), 53 virus-negative cases with thrombocytosis and 32 virus-negative cases with normal platelets. There was no statistically significant difference in sex distribution (P > 0.05). Two patients had thrombocytopenia. Except for three patients with platelets > 1000 × 10⁹/L who were orally treated with dipyridamole, the rest of the patients had their platelet counts returned to normal spontaneously.

The overall viral positivity rate was 56.5% (113/200). The virus detection rate in the thrombocythemia group (61.0%) was significantly higher than that in the normal platelet count (48.4%, P < 0.05). In the thrombocythemia group, RSV had the highest detection rate (36.5%), followed by IFV-A (21.8%) and HRV (20.6%). The detection rates of RSV and IFV-A were significantly higher than those in the normal platelet group (P < 0.05).

### 3.2 Comparison of clinical data and laboratory indices

In thrombocythemia cases, the duration of hospital stay, fever duration, cough duration, and incidence of wheezing symptoms was significantly higher in the respiratory virus-positive group than in the virus-negative group (P < 0.05). The levels of leukocytes and IL-6 were also significantly higher (P < 0.05), whereas there was no significant difference in PCT and CRP levels between the two groups (P > 0.05) (Table 1).

**Table 1:** Comparison of clinical data and laboratory indices of correlation between respiratory viruses and platelets.

variable	Virus Positive Thrombocythemia Group (n = 83)	Virus Negative Thrombocythemia Group (n = 53)	Virus Positive Normal Platelet group (n = 30)	t1/x^2^	P1	t2/x^2^	P2
Length of hospitalization (d)	6.57 ± 2.23	5.03 ± 1.13	6.30 ± 2.75	3.35	0.001	0.054	0.95
Length of fever (d)	3.97 ± 3.40	1.96 ± 1.85	2.01 ± 2.65	2.80	0.007	2.28	0.03
Length of cough (d)	8.30 ± 4.31	5.73 ± 3.15	5.01 ± 4.21	2.63	0.01	2.07	0.04
wheezing (n,%)	49 (59.04)	11 (20.75)	8 (26.67)	19.23	0.000	23.59	0.000
Non-respiratory Symptoms^*^ (n,%)	26 (31.33)	10 (18.87)	7 (23.33)	2.58	0.11	0.68	0.41
Hb (g/L)	102.07 ± 12.87	101.43 ± 9.10	103.52 ± 10.23	0.22	0.82	0.19	0.75
WBC (×109/L)	11.36 ± 3.64	8.59 ± 2.78	8.34 ± 5.03	3.31	0.002	1.96	0.06
IL-6 (pg/ml)	10.91 ± 18.11	3.95 ± 3.74	5.57 ± 4.78	2.06	0.04	1.28	0.21
CRP (mg/L)	6.12 ± 7.54	12.01 ± 15.99	5.64 ± 6.32	1.82	0.07	1.95	0.11

* t1 and P1: Comparison of clinical data between respiratory virus-positive and virus-negative groups in thrombocythemia cases; t2 and P2: Comparison of clinical data between thrombocythemia and non-thrombocythemia groups in virus-positive cases;

* Non-respiratory symptoms include myocardial damage, anemia, diarrhea, rash, and convulsions

Among the virus-positive cases, the duration of fever, cough, and incidence of wheezing symptoms were significantly higher in the thrombocythemia group than in the non-thrombocythemia group (P < 0.05), but there was no significant difference in the laboratory indices (P > 0.05) (Table 1).

### 3.3 Relationship between platelets and cough with wheeze in respiratory virus-positive infections

Among the virus-positive cases, we found that higher platelet counts were significantly associated with longer hospitalization duration (p < 0.05), prolonged cough duration (p < 0.05), and a higher co-occurring wheezing rate (p < 0.05) (Table 2).

**Table 2:** Comparison of clinical data of correlation between different degrees of thrombocythemia and its symptoms.

variable	Mild Thrombocythemia Group (n = 69)	Moderate and Severe Thrombocythemia Group (n = 14)	t/x^2^	P
Length of hospitalization (d)	6.28 ± 2.46	7.82 ± 2.04	2.97	0.037
Length of fever (d)	3.18 ± 1.51	4.22 ± 2.10	2.77	0.12
Length of cough (d)	7.87 ± 3.43	8.96 ± 2.54	3.14	0.025
wheezing (n,%)	37 (53.62)	12 (85.71)	4.97	0.026
Non-respiratory Symptoms^*^ (n,%)	19 (27.54)	7 (50.0)	2.73	0.098

* Non-respiratory symptoms include myocardial damage, anemia, diarrhea, rash, and convulsions

Point-biserial correlation analysis showed that thrombocythemia was closely related to respiratory virus positivity and was complicated by cough and wheezing (r = 0.314, P < 0.05).

### 3.4 Logistic regression analysis of influencing factors

Multifactorial logistic regression analysis indicated that age, non-respiratory symptoms, platelet levels, and IL-6 level were independent factors influencing the development of cough and wheezing in respiratory virus-positive children (P < 0.05) (Table 3).

**Table 3:** Logistic regression analysis of factors influencing the complication of cough and asthma in respiratory virus positive patients.

variable	B	Wals	P	OR	95%CI
age	−0.164	1.683	0.021	0.768	0.515-1.144
Length of hospitalization	0.131	0.173	0.678	1.14	0.615-2.113
Length of fever	0.097	0.323	0.683	0.997	0.981-1.013
Non-respiratory Symptoms*	3.256	5.762	0.016	25.933	1.817-370.03
WBC	−0.069	0.566	0.452	0.933	0.779-1.118
CRP	0.028	0.029	0.865	1.029	0.742-1.426
IL-6	0.102	1.736	0.039	1.118	0.867-2.442
PCT	4.502	0.734	0.392	57.499	0.005-6106
constant	−0.6551	0.014	0.090	0.619	–

### 3.5 Value of platelets in assessing hospital stay and cough duration

**Fig 1:**
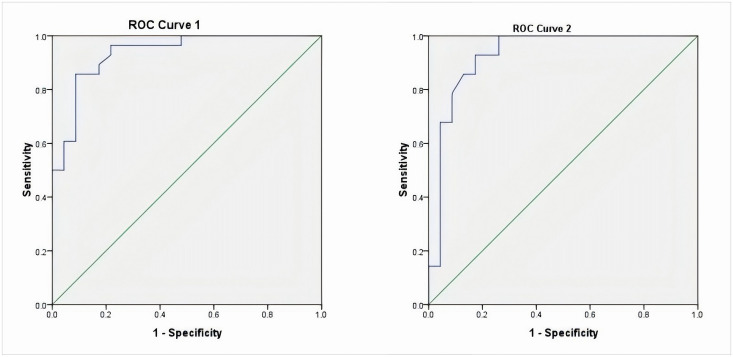
ROC curve of platelets in assessing hospital stay and cough duration. ROC curve 1 of hospitalization time for respiratory virus-positive infants evaluated by platelet count; ROC curve 2 for assessing cough and wheezing time in respiratory virus-positive infants using the platelet assay.

ROC curve analysis demonstrated that the areas under the curve of thrombocythemia for assessing hospital stay duration and cough duration were 0.964 and 0.936, respectively (P < 0.05), indicating a significant assessment value (Fig 1).

## 4. Discussion

Respiratory viral infections are prevalent in infants and may present with different clinical manifestations, usually without special treatment; however some children often present with significant coughing and wheezing, or even shortness of breath. The most common viruses include respiratory syncytial virus, influenza A and B viruses, rhinovirus, adenovirus, and EBV [9]. In this study, we found that respiratory syncytial virus was the most common respiratory virus infecting infants in Anqing, accounting for 36.5% of the cases, followed by influenza A and rhinovirus, which are similar in most regions [9,10]. Respiratory viruses-induced cough and wheezing in infants are clinically frequent, and may be due to their influence on the development of cough and wheezing through immune damage, airway epithelial barrier damage, inflammatory infiltration of the airways, and airway hyper-responsiveness. Studies have shown that infant wheezing is closely related to respiratory viral infections, secondary thrombocytosis was more likely to occur in younger patients who had clinical manifestations of wheezing and dyspnea and who had been diagnosed with bronchiolitis [11]. In this study, we also found that age, non-respiratory symptoms, platelets, and IL-6 were the main factors influencing the development of cough and wheezing in respiratory virus-positive infants. The younger the age, the more non-respiratory symptoms, and the higher the platelet and IL-6 levels, the higher the probability of cough and wheezing in infants.

Secondary thrombocytosis is frequently observed in infants, young children, and neonates. In general, markedly elevated platelet counts may generally result in hypercoagulability and, in some cases, thrombosis in affected pediatric patients. However, thrombocytosis in infants is predominantly benign and often resolves spontaneously without intervention, which contrasts with the condition in adults. Hemorrhagic and thromboembolic complications are rarely associated with infantile thrombocytosis [12]. Infantile thrombocythemia is categorized as either primary or secondary. Primary hereditary thrombocythemia may be caused by germline mutations in the gene encoding a key regulator of thrombopoiesis thrombopoietin (THPO) and its receptor c-MPL (MPL) or the receptor effector kinase Januskinase2 (JAK2) [11]. Studies have shown that infantile thrombocythemia is mostly secondary and generally associated with infection, tissue ischemia organ damage, immune abnormalities, trauma, etc., and mostly with viral infections [13], which is consistent with the higher incidence of thrombocythemia in respiratory virus-positive children in this study. Studies have reported that small infants with a diagnosis of capillary bronchiolitis and clinical manifestations of wheezing are more likely to develop ST and have a longer duration of illness, thrombocythemia may be a retrospective marker of the respiratory inflammatory response following viral infection and may indicate the severity of respiratory infection [7,8]. In this study, we found that among the virus-positive cases, the duration of fever and cough in the thrombocythemia group was higher than that of the children in the platelet-normal group, and the combined wheezing symptoms of the children were significantly higher than those of the platelet-normal group, suggesting that thrombocythemia can be used as an indicator of the prognosis of the disease in infants, which needs to be considered by clinicians. However, the mechanism of the cause of platelet increase due to viral infection is unclear. Thrombopoietin (TPO) is a key regulator in the process of platelet production, which binds to TPO receptors on megakaryocytes, and then activates megakaryocytes through a variety of signaling pathways, prompting megakaryocytes to undergo proliferation and differentiation thus producing platelets [14,15]. During infection or inflammation, elevated levels of TPO levels stimulate megakaryocytogenesis, leading to platelet production. It has been shown that ST is associated with the physiologic metabolism of platelet basal values and TPO in infancy and childhood, especially in the neonatal period, which is the physiologic peak of thrombopoietin that declines with age [3,16]. It has been suggested that high expression of TPO and its associated receptors and elevated IL-6 levels in children with concomitant viral infections are associated with thrombocytosis [17]. However, in our study, we found that in respiratory virus-positive children, there was no significant difference between the thrombocythemia group and the platelet-normal group in terms of IL-6, PCT, and WBC, suggesting that further studies are needed to determine whether or not thrombocythemia and inflammatory mediators are related in children with viral infections. As discussed above, age, non-respiratory symptoms, platelet levels, and IL-6 were identified as independent influencing factors. Younger age might render infants more vulnerable to respiratory viral infections, and non-respiratory symptoms could be an indicator of a more severe systemic response. Moreover, elevated platelet levels and IL-6 may be involved in the inflammatory process, thus promoting the development of cough and wheezing.

The level of platelet elevation may serve as a reference for the duration of cough and wheezing and the length of hospitalization. Studies have reported that children with ST due to pneumonia from viral infections may exhibit more severe clinical symptoms and extrapulmonary complications, which should be emphasized by clinicians [18].We found moderate-to-severe thrombocytosis was significantly associated with prolonged wheezing in the virus-positive infants, suggesting a potential role in airway inflammation.Our ROC curve analysis showed that the hospitalization time and duration of cough were longer in respiratory virus-infected children with thrombocythemia, and the surface thrombocythemia has a certain value in evaluating the hospitalization time and duration of cough in respiratory virus-positive children, suggesting that the clinic can initially screen infants at high risk of cough by using the peripheral blood PLT level, and further examination and diagnosis for the high-risk group may be helpful for saving medical resources and improving the estimated value. This may be of great significance in saving medical resources, improving the accuracy of the assessment results, and promoting the development of precision medicine.

## 5. Conclusion

Infant respiratory viruses are closely associated with secondary thrombocythemia, and elevated platelet levels increase hospitalization and prolonged cough and wheezing in infants, which can be assessed early to predict the risk of the infant and reduce anxiety and stress in the parents.
